# Loot

**Published:** 1873-07

**Authors:** 


### Loot.

### *Dengue or Break-Bone Fever.*

From the third of September till the twenty-ninth of November, 1872, I had occasion to treat one hundred and twenty-seven cases of Dengue Fever. Nearly all the cases were of a mild type; only two or three required treatment in the hospital. Before the fever made its appearance at this place, it had been very prevalent both at Matamoras, Mexico, and Brownsville, Texas, towns adjoining this post. None escaped it; old and young, natives and strangers, were alike attacked, as well as many old inhabitants who had escaped during former visitations. There were no deaths from the fever, either in the towns or the garrison. The type, as it appeared among the troops, was milder than that in the towns adjoining. This may be due in some measure to the mild treatment I used, and the more heroic treatment adopted by the civil physicians.

My plan of treatment was rest, low diet, hot foot baths when the fever was high, at bedtime small doses of a mixture of fluid extract of colchicum, antimonial wine, spirits nitre and nitrate of potash, repeated every six or eight hours till the colchicum began to affect the bowels. After this I gave no more medicine for a day or two, until the end of the period of fever, which lasted generally seventy-two hours; when I gave five to eight grains of quinine in solution three times a day, till the system was fully under the effects of the medicine. Average length of time for each case under treatment, 4.29 days.

I am fully convinced from observation and from personal experience, as my own was the first case of the fever in the garrison, that the Dengue is an indigenous yellow fever, running a course precisely similar to yellow fever (of which disease I had an attack about two years ago), the fever lasting forty-eight or seventy-two hours, with increased temperature in the evening, the period of calm of longer or shorter duration, terminating often in a profuse sweat, having a characteristic odor very similar to the transudations in yellow fever, followed by a period of convalescence which is generally short and rapid, but sometimes slow, and always attended with a good deal of pain in the bones. The symptoms and signs are the same as in yellow fever, only less marked: pain in the back of the neck and loins, congested eyes, but rarely ferrety as in yellow fever, tongue velvety, with red tip and edges, the attack coming on suddenly, in almost every case in the midst of the most robust health. The disease spreads by the action of fomites, like yellow fever, and is not contagious.

I had no opportunity to test the axillary temperature, for the reason that my thermometer got broken in transportation; but in no case was the skin very hot, so far as could be judged by applying the hand to the surface. Thirst was not very troublesome; after the fever there was great indisposition to sleep for several nights, in some cases requiring an anodyne at bedtime.—*W. E. Whitehead, M.D., U.S.A., in Pacific M. & S. Journal.*

### *Cod Liver Oil and Lacto-Phosphate of Lime.*

This remedy is being quite extensively prescribed by physicians, and as considerable inquiry has been made as to an eligible mode of prescribing it, I will give my experience in the manufacture of the article, and also a simple process for making syrup of lacto-phosphate of lime.

For a long time I have had demand for a tasteless cod liver oil, and have been in the habit of preparing it in the form of an emulsion with gum arabic and water, and covering the odor with a few drops of essential oil of bitter almonds.

Over a year ago I found physicians were prescribing cod liver oil and lacto-phosphate of lime, and I devised a formula for it, based on my experience with the simple emulsion and the syrup of lacto-phosphate of lime, for which a considerable demand had sprung up. The formula I then devised has been followed by me up to the present time, and has invariably given satisfaction, and produces an article which does not separate or become rancid.

I think, however, it should be prepared extemporaneously as prescribed by physicians, and I have not kept it on hand, but prepare it as wanted, thus always giving a perfectly sweet article.

Take of gum arabic, oz.ij dr.ij; water, f oz.ij; syrup lacto-phosphate of lime, f oz.vi; cod liver oil, f oz.viij; essential oil bitter almonds, six drops; rub the gum, water and syrup together, until a smooth mucilage is made, then add the oil gradually with constant stirring, and, lastly, the oil of bitter almonds.

Thus made, each table-spoonful of cod liver oil and lacto-phosphate of lime contains four grains lacto-phosphate of lime and fifty per cent. of cod liver oil. The gum in the above should be selected, ground and passed through a sieve of sixty meshes to the inch. Cod liver oil and lacto-phosphate of lime prepared in this manner, forms a preparation free from unpleasant taste and odor, and enables the practitioner to administer these valuable remedies without repugnance on the part of the patient.

*Syrup Lacto-phosphate of Lime.*—Take of chloride of calcium, oz.i; phosphate of soda, oz.iv; concentrated lactic acid, oz.i; dissolve the chloride of calcium and phosphate of soda separately, and mix the solutions; wash the precipitate and dissolve in the acid. Filter and mix with sufficient syrup to make two and one-half pints.—*Edward Chiles, in American Journal of Pharmacy.*

### *Treatment of Nervous Aphonia and Chronic Pharyngitis.*

Dr. Mandl, in his "Traite Pratique des Maladies du Larynx et du Pharynx," quoted in the Dublin *Journal of Medical Science*, April, 1873, notes as an important clinical fact, that an essential nervous aphonia, viz., bilateral dynamic paralysis of the tensors of the vocal cords (crico-thyroideans) may perhaps be in young girls the precursor of a tubercular inflammation which declares itself later. The return of the voice on the application of electricity is not an absolute security. It is in such cases especially that it is necessary to abstain, according to Trousseau, from the employment of ferruginous preparations, which determine a sanguineous plethora by no means devoid of serious inconveniences in individuals predisposed to hæmoptysis and to tubercularization.

For a long time Dr. Mandl also has prescribed the use of iron in chronic laryngitis and pharyngitis, as the plethora consecutive to its administration turns into a local hyperæmia; consequently, chronic phlegmasias are more often kept up by it than amended.

That particularly troublesome complaint, known as granular (follicular) pharyngitis, or clergymen's sore-throat—generally chronic in its nature, and, though often temporarily relieved, apt to relapse—Dr. Mandl has succeeded in curing, by painting the granulations twice a day, with a solution composed of one part of metallic iodine and one of carbolic acid, dissolved, by means of iodide of potassium, in one hundred parts of glycerine. If irritation supervene, the application is less frequently applied, or superseded for a time. The largest granulations are first scarified, and then touched with the glycerole, but in a more concentrated form, and in variable proportions, according to the degree of the affection. This local treatment alone is, he believes, sufficient to radically cure the disease, independently of any supposed diathesis. Dr. Mandl may probably have been led to adopt this mode of treatment from Dr. Hastings, who recommended the application to the "mucous crypts which had previously resisted the remedial effects of nitrate of silver," of a "saturated solution of iodine in rectified spirit."

### *Why Insanity Increases.*

Sir James Coxe, Commissioner of Lunacy for Scotland, in discussing this subject, strikes at the root of this and many another evil of our civilized life, when he says in his last address:—

"Communities should be trained in a knowledge of the human organism, and of the laws which determine its welfare. Ignorance of such knowledge has an all-pervading influence. It affects the proceedings of the legislature, of the clergy, and of teachers, and through their instrumentality the conduct and behavior of the whole community. In the first place, the complex nature of the human mind is overlooked; education is too much restricted to the cultivation of the intellectual faculties, and even their training is, as a rule, only partial and imperfect. In the second place, moral training may be said to be almost entirely neglected; and the same remark is applicable to physical training.

"The neglect of physical training is almost universal, and even where it is attempted, it is calculated to do, perhaps, more harm than good.

"My doctrine then is, gentlemen, that insanity, so far from being a disease of civilization, is a disease of ignorance, and that the only way in which its extension may be checked is by imparting to every man a knowledge of the structure of his own body, and of the relations in which he stands to the moral and physical world around him."

This is sound doctrine, and we commend it to the thoughtful consideration of that class of physicians who fancy any physiological instruction furnished the public is *margarita ante porcos.*—*Philadelphia Reporter.*

### *A Physician's Diary of Business.*

A pocket diary has been picked up in the street, and now is in the finder's possession, awaiting its owner. From the following extracts, it appears the loser was a medical man:

"Kase 230. Mary An Perkins. Bisnes, washwoman. Sikness in her hed. Fisik sum blue pils a soaperifik; age 52. Ped me one dollar, 1 kuarter bogus. Mind get good kuarter and mak her tak mo fisik.

"Kase 231. Tummes Krinks, Bisines, Nirishman. Lives with Pady Molouny whot keeps a dray—Sikness, digg in ribs and tow blak eys. Fisik to drink my mixter twict a day of sasiperily bere and jellop, and fish ile, with asifidety to make it taste fisiky. Rubed his face with kart grese liniment, aged 39 years of age. Drinked the mixter and wuddnt pay me bekase it tasted nasty, but the mixter'll work his innards, I reckon.

"Kase 232. Old Misses Boggs. Aint got no bisnes, but plenty of money. Siknes awl a humbug. Gav her sum of my celebrated "Dipseflorikon," which she sed drank like cold tee—wich it was too. Must put sumthink in it to mak her feel sik and bad. The Old Wommen has got the roks."

***DeWitt County Medical Society.***

The DeWitt County Medical Society met in annual session on the 29th day of April, 1873.

Members present—Drs. J. H. Potter, T. W. Davis, John H. Tyler, John Wright, Z. H. Madden, Thomas K. Edmiston, and Christopher Goodbrake.

Also present—Dr. J. J. Starkey, of Waynesville, and Dr. W. H. Crothers, of Maroa, Macon county.

Dr. Potter, President, in the chair.

The minutes of the last meeting were read and approved.

On motion of Dr. J. Wright, Dr. W. H. Crothers was elected an honorary member of the society.

Dr. Wright proposed Dr. J. J. Starkey for membership, and the Censors having reported favorably on his case, he was, on motion of Dr. Goodbrake, elected a member of the society.

The election of officers being in order, the following gentlemen were elected for the ensuing year: President—Dr. J. H. Tyler, DeWitt; Vice-President—Dr. T. W. Davis, Wapella; Secretary—Dr. C. Goodbrake, Clinton; Treasurer—Dr. Z. H. Madden, Clinton. Censors — Dr. W. G. Cochran, Farmer City; Dr. J. H. Potter, Wapella; Dr. J. J. Starkey, Waynesville.

Dr. Tyler, on taking the chair, delivered a short address, which was well received by the members present.

Dr. Thomas W. Davis reported the case of a gentleman in whom very strange symptoms had occurred from the administration of an enema, consisting of a tablespoonful of common table salt in solution.

Dr. Tyler reported three very interesting cases of *cerebro-spinal meningitis.*

Diseases of the Liver was chosen as the subject for discussion at the next meeting.

The essayists having failed to perform their duties, they were, by order of the President, continued.

On motion of Dr. Goodbrake, the following resolution was unanimously adopted:

*Resolved,* That the thanks of the society are due and are hereby tendered to the editors of the *Clinton Public* and *Clinton Register* for kindly publishing notices of meetings of this society.

On motion of Dr. Wright, the thanks of the society were tendered to the proprietors of DeWitt Hall for inviting the society to meet in their splendid room without any pecuniary remuneration.

On motion, the Secretary was ordered to furnish copies of the proceedings of this meeting for publication in the Chicago medical journals, our county papers, and the *Maroa News.*

On motion of Dr. Madden, the society adjourned to meet in quarterly session in Clinton, on the first Tuesday of July next.

C. Goodbrake, M.D., *Sec.*

